# Space-Dependent Formation of Central Pair Microtubules and Their Interactions with Radial Spokes

**DOI:** 10.1371/journal.pone.0110513

**Published:** 2014-10-21

**Authors:** Yuki Nakazawa, Tetsuro Ariyoshi, Akira Noga, Ritsu Kamiya, Masafumi Hirono

**Affiliations:** 1 Department of Biological Sciences, Graduate School of Science, University of Tokyo, Bunkyo-ku, Tokyo, Japan; 2 Department of Life Science, Faculty of Science, Gakushuin University, Toshima-ku, Tokyo, Japan; Cancer Research UK London Research Institute, United Kingdom

## Abstract

Cilia and flagella contain nine outer doublet microtubules and a pair of central microtubules. The central pair of microtubules (CP) is important for cilia/flagella beating, as clearly shown by primary ciliary dyskinesia resulting from the loss of the CP. The CP is thought to regulate axonemal dyneins through interaction with radial spokes (RSs). However, the nature of the CP-RS interaction is poorly understood. Here we examine the appearance of CPs in the axonemes of a *Chlamydomonas* mutant, *bld12,* which produces axonemes with 8 to 11 outer-doublets. Most of its 8-doublet axonemes lack CPs. However, in the double mutant of *bld12* and *pf14*, a mutant lacking the RS, most 8-doublet axonemes contain the CP. Thus formation of the CP apparently depends on the internal space limited by the outer doublets and RSs. In 10- or 11-doublet axonemes, only 3–5 RSs are attached to the CP and the doublet arrangement is distorted most likely because the RSs attached to the CP pull the outer doublets toward the axonemal center. The CP orientation in the axonemes varies in double mutants formed between *bld12* and mutants lacking particular CP projections. The mutant *bld12* thus provides the first direct and visual information about the CP-RS interaction, as well as about the mechanism of CP formation.

## Introduction

Motile cilia and flagella are ancient organelles present in various eukaryotic organisms including humans. Defects in structure or motility of cilia and flagella result in a category of diseases called primary ciliary dyskinesia [1], [2]. The axoneme of motile cilia and flagella has a strikingly conserved structure consisting of nine outer doublet microtubules and two central-pair (CP) microtubules. These microtubules have various types of projections, such as outer and inner dynein arms, radial spokes (RSs), CP projections and nexin links, which directly or indirectly interact with each other [3]–[8]. The interactions between these projections must play fundamental roles for maintaining the cylindrical arrangement of the outer doublets, for producing the motive force, and for converting the force to ciliary/flagellar bending waves [9]–[11]. However, the nature of interaction between these structures is not well understood.

The ninefold symmetrical arrangement of the outer doublets is determined by the centriole (basal body), which has a cylindrical shape consisting of nine short triplet microtubules. Axonemal doublet microtubules assemble onto the A- and B-tubules of each triplet microtubule through a junction called the transition zone [11]. In contrast to the outer doublets assembling on clearly defined template structures, the assembly process of the CP is largely unknown. In *Chlamydomonas* flagella, the distal end of the CP is capped with a plate while the proximal end does not attach to any recognizable structure [12], [13]. In *Tetrahymena* cilia, the distal end is also capped with a plate, while the proximal end of one CP microtubule is covered with the axosome, an amorphous structure observed in ciliate axonemes, and the other CP microtubule is apparently associated with no distinct structures [14], [15]. Neither of the ends appears to function as the nucleation site for CP assembly [16]–[20]. However, what nucleates the CP assembly and what determines the CP number in the axoneme are not known.

The CP and RS appear to function as a regulatory system for ciliary and flagellar motility [21]–[25]. For generation of axonemal beating, a subset of the axonemal dyneins must be activated at a specific phase of beating, and the region of the activated dynein must move along the axoneme as the bending propagates toward the tip. Although the mechanism of this dynein regulation is yet to be elucidated, the CP/RS system clearly plays a crucial role in coordinating dynein activities [9], [10], [23]. Several lines of evidence suggest that, in some protists including *Chlamydomonas* and *Paramecium*, the CP assumes a twisted conformation and rotates within the axoneme once per beating cycle. The signal of CP orientation is most likely transmitted to the dynein arms through the RS [9], [10], [26]–[28]. The CP-RS interaction probably involves mechanical force since the RS is the structure that keeps the CP at the center of the axoneme [24]. In accordance with the probable mechanical nature of interaction, a recent study has suggested that the RS pushes the CP in beating axonemes [29]. However, the evidence is rather indirect and thus whether the RS pushes or pulls the CP appears to need further studies.

We previously reported a *Chlamydomonas* mutant, *bld12*, that has severe defects in a subcentriolar structure termed the cartwheel [30]. The cartwheel, consisting of a central hub and nine spokes, is located at the proximal end of the centriole as a stack of several layers [31], [32]. The mutant *bld12* lacks the central part of the cartwheel due to a null mutation in the gene coding for SAS-6, a component of the cartwheel [30]. X-ray crystallography and biochemical analyses showed that SAS-6 forms a dimer having two globular heads and a coiled coil tail, and the dimers assemble into a ring through their hydrophobic interaction between the heads [33], [34].

Lack of the cartwheel in *bld12* causes severe defects in the centriole assembly: ∼80% of the centrioles observed are split into fragments and only ∼20% are assembled in the cylindrical structure. Interestingly, the number of the triplets varies from seven to eleven in the cylindrical centrioles. As a consequence, flagellar axonemes produced in a small fraction of *bld12* cells contain variable numbers of outer doublet microtubules ranging from eight to eleven [30]. In this study, we investigated the effects of the variation in the outer doublet number on the appearance of the CP within the axoneme. The results revealed the importance of the spatial factor for the formation of the CP, and furthermore, provided evidence for the presence of attractive force between the CP and RS.

## Material and Methods

### Strains

*Chlamydomonas* strains CC124 (wild-type), *pf6*, *pf14*, and *pf16* were obtained from the *Chlamydomonas* Resource center, and *cpc1* from Dr. Mitchell of State University of New York Upstate Medical University [35]. The mutant *bld12* (*bld12-1*) was previously isolated in our laboratory (Nakazawa et al., 2007). The double and triple mutants, *bld12pf6*, *bld12pf14*, *bld12pf16*, *bld12cpc1*, *bld12cpc1pf6*, *pf14pf6*, *pf14pf16,* and *pf14cpc1*, were produced by genetic crosses [36]. Cells were grown in Tris-acetate-phosphate (TAP) media [37] with aeration on a 12 h/12 h light/dark cycle, or under constant illumination with agitation.

### Preparation of flagellar axonemes

Flagella were isolated from *bld12-1* or *bld12-1cw92* cells by the dibucaine method of Witman [38]. Detached flagella were collected by centrifugation at 10,000×g, overlaid on a sucrose cushion (25% sucrose, 20 mM HEPES pH 7.4) and centrifuged at 1,000×g for 10 min at 4°C. Flagella at the boundary between the upper phase and the sucrose solution was collected, and demembranated by treatment with 0.5% Nonidet P-40 in HMDE solution (30 mM HEPES, 5 mM MgCl_2_, 1 mM dithiothreitol (DTT), 1 mM EGTA, pH 7.4) [38]. The axonemes collected by centrifugation were resuspended with HMDE solution. For analysis of flagellar length, isolated flagella were collected and their lengths were measured using ImageJ software.

### Electron microscopy

Axonemes were prefixed with 2% glutaraldehyde and 1% tannic acids in 50 mM phosphate buffer pH 7.2 for 1–2 h at 0°C, and postfixed with 1% OsO_4_ in the phosphate buffer for 1 h. The samples were dehydrated by passing through graded concentrations of alcohol solutions, and embedded in EPON 812. Thin sections (50–70 nm) were stained with aqueous uranyl acetate and Reynold’s lead citrate, and observed with a JEM1010 electron microscope. Images were obtained using a film-base camera or MegaView III digital camera (JEOL, Tokhyo). For analysis of axonemal cross sections, images were chosen on the basis of clear appearance of all outer doublets, to ensure that the axoneme under examination was cut almost normal to the axoneme axis. The diameter and inner-doublet spacing were measured using ImageJ software. Correlation between the diameter and the doublet number was analyzed using a linear regression model. Group difference in inter-doublet spacing was analyzed using ANOVA. Statistical significance for the test was set at P<0.05.

### Analysis of CP-RS association and helical properties of the CP

For analyses of the CP-RS association, cross section images of 10- or 11-doublet axonemes of *bld12*, *bld12pf6*, *bld12cpc1*, *bld12cpc1pf6*, and *bld12pf16* were chosen and collected based on the clarity of the C1 and C2 microtubules. In the case of *bld12pf16*, images of the 9-doublet axonemes were used for the analysis in addition to the 10- or 11-doublet axonemes. Each image was oriented with the dynein arms projecting clockwise. The CP surface in the image, which was approximated to a circle, was equally divided into 12 sectors. The spokeheads attached to or detached from the CP were identified by visual inspection. The center of the consecutive spokeheads attached to the CP was defined as the spokehead-interaction site. Distribution of the interaction sites on the CP surface was represented by polar histograms.

In the CP orientation analysis in cross section images of *pf14*, *pf14pf6*, *pf14cpc1*, *pf14pf16* axonemes, distribution of the sector closest to the outer doublet wall was represented by polar histograms. Statistical significance of the difference in distribution was evaluated by the χ^2^ test. Statistical significance was set at P<0.05.

## Results

### Abnormal axonemes in *bld12*

**Figure 1:**
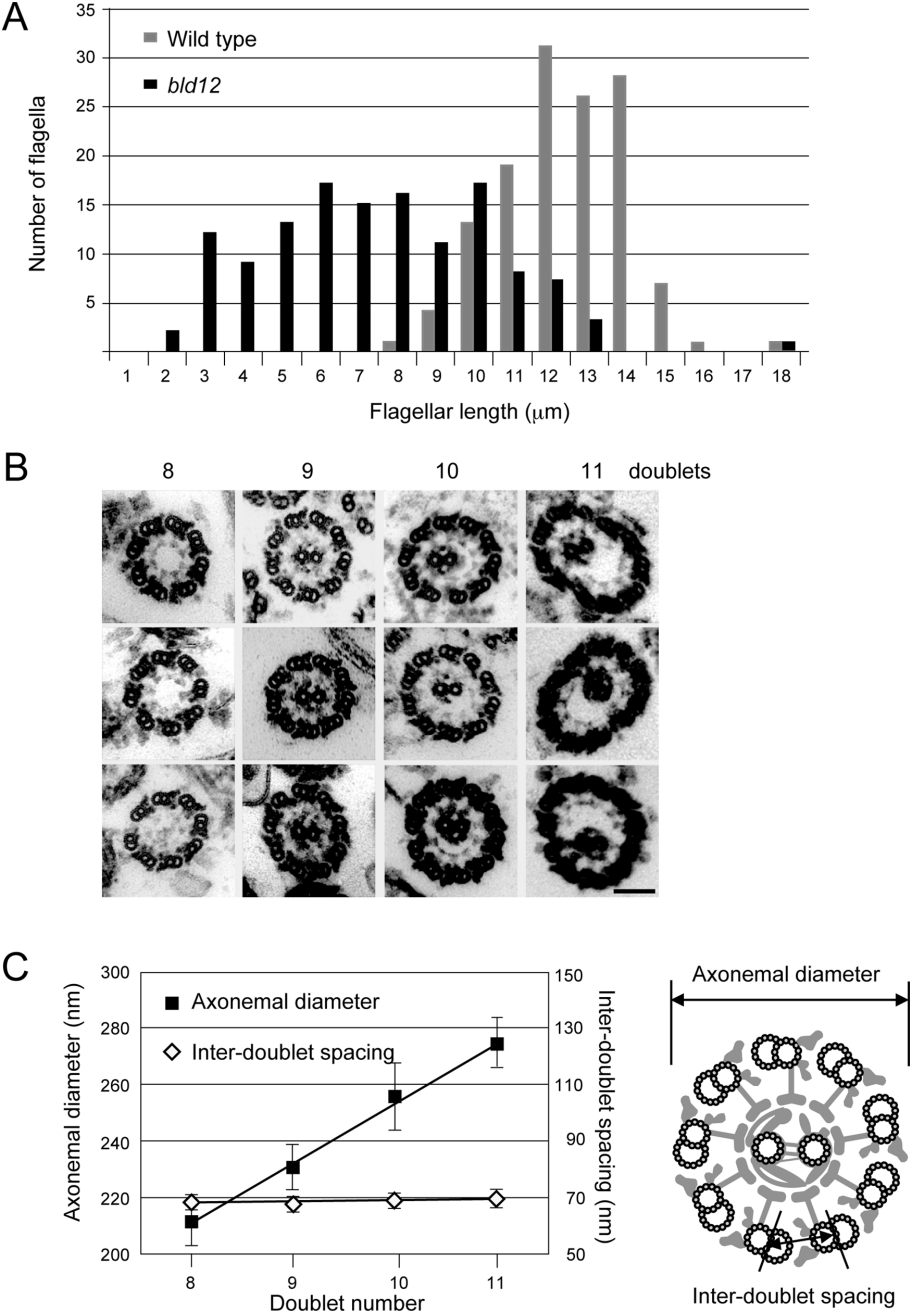
Abnormal features of *bld12* axonemes. (A) Length distributions of wild type (gray) and *bld12* (black) flagella. (B) Axonemes with 8, 9, 10, and 11 outer doublet microtubules. Bar, 100 nm. (C) Diameter and inter-doublet spacing in axonemes with various doublet numbers. Diameter increases with the doublet numbers, while the inter-doublet spacing is constant. The linear regression slope of the diameter is 22.14±1.69 nm (R^2^ = 0.900). The difference between the inter-doublet spacing is not significant (ANOVA, P = 0.59).

As we previously reported, ∼10% of *bld12* cells produced one or two flagella when cell walls were removed by treatment with autolysin [30]. Under this condition, about 8% of cells were uniflagellated and 2% biflagellated. Of the biflagellated cells, about 50% had flagella of unequal lengths. The flagellar length was variable but always shorter than that of wild type (Figure 1A). The *bld12* flagella displayed a variety of motility phenotypes, ranging from complete paralysis to sporadic twitching to almost normal beating.

Electron microscopy showed that the percentage of 8-, 9-, 10-, and 11-doublet axonemes was, respectively, ∼5%, ∼90%, ∼5%, and ∼0.1% (Figure 1B) [30]. While the diameter of the axoneme increased with the doublet number, the space between the adjacent doublets was constant (Figure 1C), suggesting that the inter-doublet structures such as the inner- and outer-dynein arms and the nexin links were not distorted in the abnormal axonemes. In the images of these axonemes, we noticed two remarkable features that are not seen in normal axonemes: ∼95% of the 8-doublet axonemes had no CP microtubules; and, in 10- or 11-doublet axonemes, the circular arrangement of the doublets was distorted because only three to five RSs were attached to the CP. Similarly distorted axonemes were also observed in intact flagella of this mutant (Yuki Nakazawa, unpublished observation).

### Effects of RS removal on CP formation

**Figure 2:**
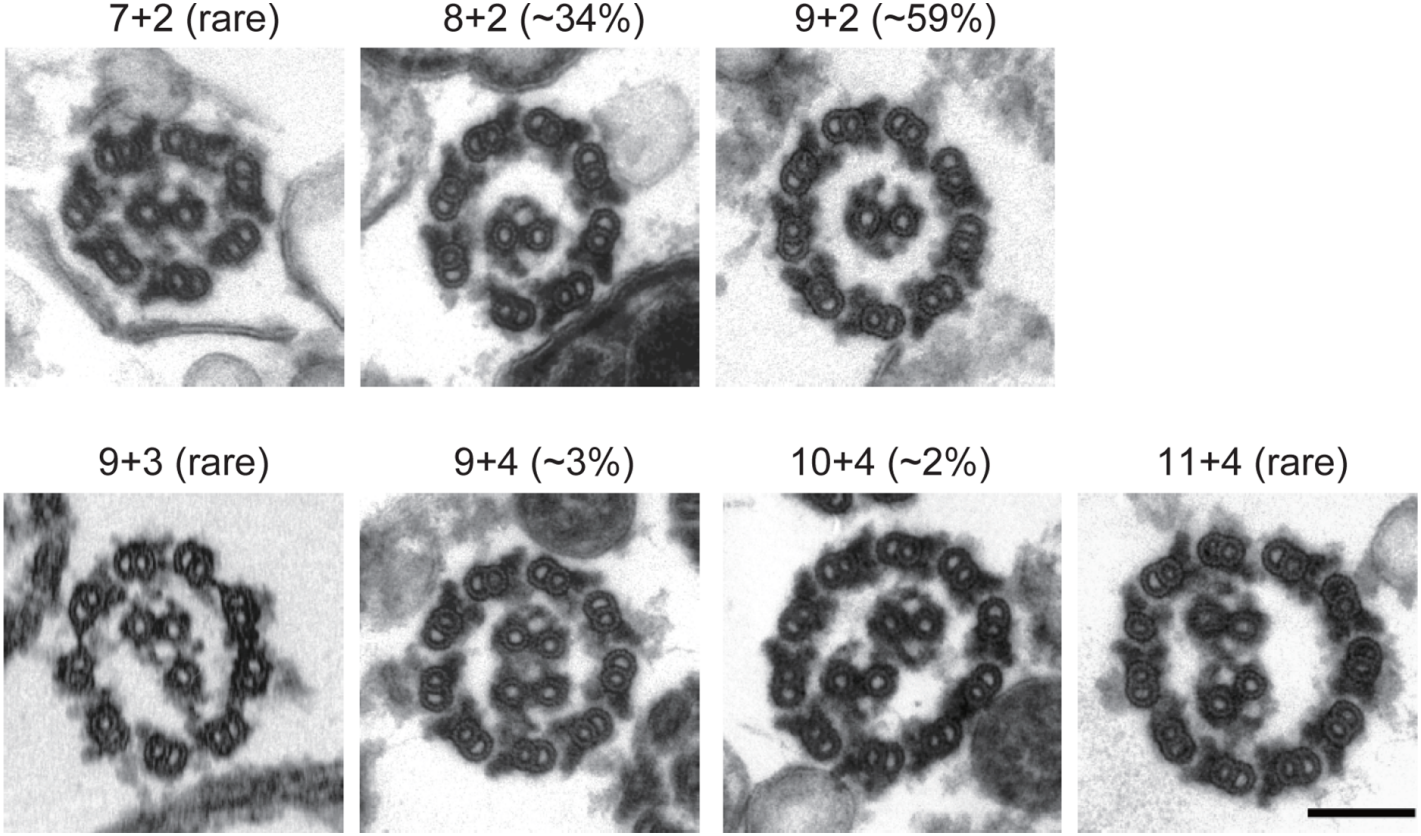
CPs in RS-lacking axonemes. Cross section images of *bld12pf14* axonemes with 7 to 10 outer doublets are shown. The frequency of each pattern in the observed images is indicated in the parenthesis (n = 1,369, Table 1). All CPs have the normal polarity in the axoneme as judged by their CP projections. Bar, 100 nm.

Absence of the CP in most of the 8-doublet axonemes led us to assume that these flagella do not have enough room to accommodate a CP in the central area. To test this hypothesis, we produced the double mutant of *bld12* and *pf14,* a mutant that lacks the RSs and has a larger internal space [39]. This mutant, *bld12pf14,* also produced axonemes with a variable number of doublets, ranging from 7 to 11 (Figure 2). As expected, all of the 8-doublet axonemes and even 7-doublet axonemes had the CP (Figure 2, Table 1). In addition, ∼5% of the 9-doublet axonemes or ∼74% of the 10-doublet axonemes contained three or four central microtubules (Figure 2, Table 1). In *pf14* also, axonemes with three central microtubules were observed although the occurrence was rare (∼0.7% in 2000 cross-section images). These observations indicate that the formation of the CP depends on the size of the space limited by the RSs and the outer doublet microtubules.

**Table 1:** The number of axonemes with particular numbers of outer doublets and central microtubules observed in *bld12pf14*.

	# of central microtubules per axoneme		
# of outer doublets per axoneme	0 or 1	2	3 or 4
7	1	2	0
8	0	465	0
9	15	801	42
10	3	5	23
11	0	1	1

(Total number of axonemes counted, 1,369).

### Spokeheads preferentially bind to distinct sites on the CP surface

Cross section images of the 10- or 11-doublet axonemes of *bld12* showed that five or six doublets were tethered by RSs to the CP to form a semicircle of a normal diameter. The rest of the doublets, not tethered to the CP due to the detachment of RSs, are arranged in another semicircle bulging outward (Figure 1B). Whether an RS was attached or detached could be easily judged from the position of the bulky spokehead. The doublet arrangement was distorted at the junctions of the two semicircles. In contrast, no such distortion was observed in the RS-lacking axonemes of *bld12pf14* (Figure 2). These observations suggest that the RS binds to the CP to help the doublets align in a circular arrangement of a constant diameter, and that the CP-RS binding is strong enough to distort the arrangement in 10- or 11-doublet axonemes.

**Figure 3:**
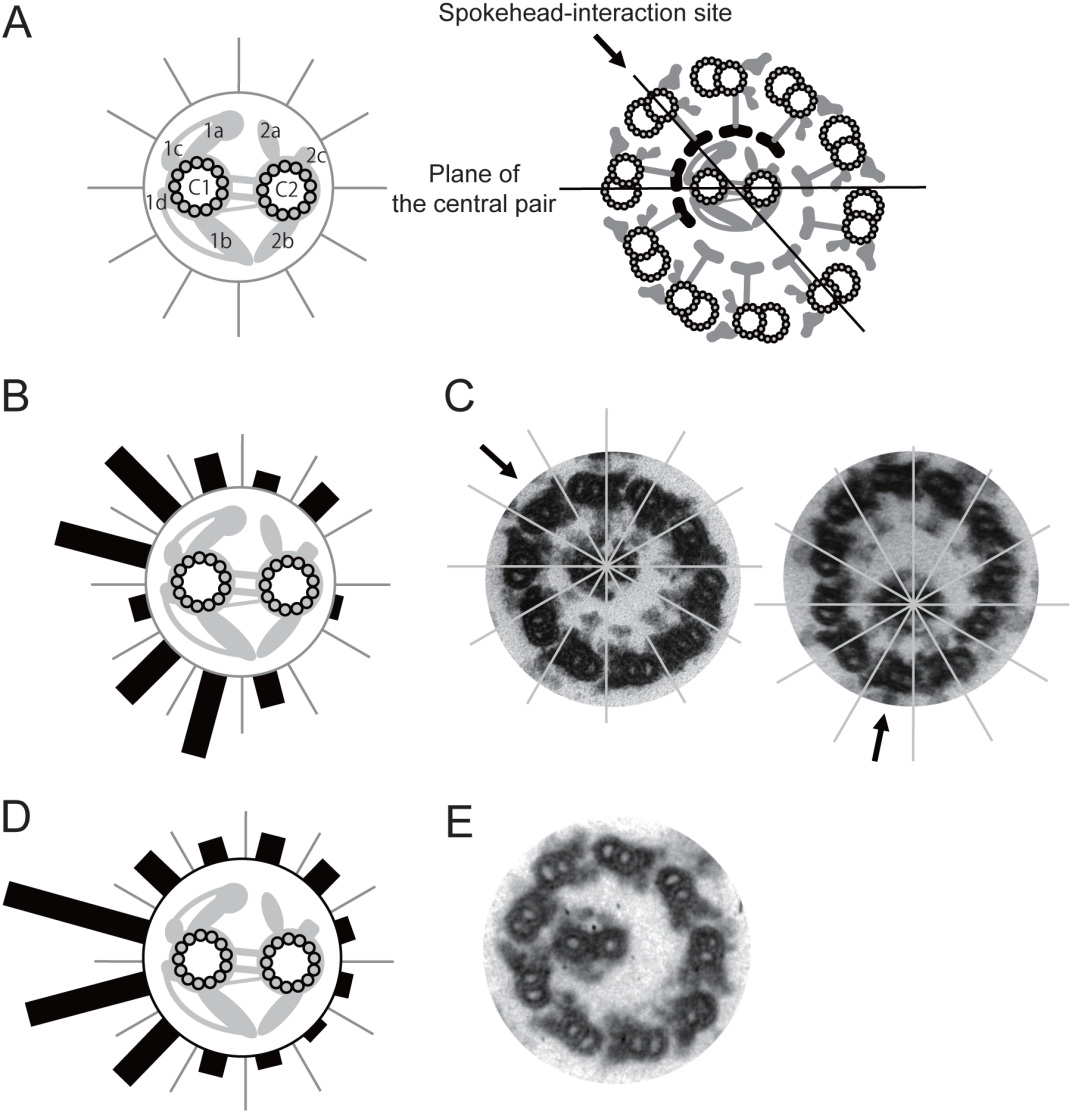
Frequency of CP-RS association in different sectors of the CPs. (A) The CP cross section was divided into 12 sectors by six lines including the one connecting the centers of the two central microtubules. The center of the consecutive spokeheads in contact with the CP was defined as the spokehead-interaction site. Major CP projections are designated [35]. (B) A polar histogram representing the distribution of the spokehead-interaction sites on the CP in cross section images of 10- or 11-doublet axonemes of *bld12* (n = 56). The length of each bar represents relative frequency to locate at the spokehead-interaction site. (C) Axoneme images that correspond to the two peaks in the histogram in (B). (D) Distribution of the CP region in contact with the wall of outer doublets in *pf14* axonemes (n = 80). The histogram suggests that the C1 microtubule is located on the outer surface on the helical CP. (E) A cross section image that represents the peak distribution in the histogram in (D). The differences between the distributions of *bld12* (B) and *pf14* (D) are significant (χ^2^ test, p<0.05).

To investigate whether this RS binding occurs on particular regions on the CP surface, we examined cross-section images of 10- or 11-doublet axonemes for the possible location on the CP where the spokeheads preferentially attach. We divided the CP image in cross section into 12 sectors, and scored the frequency of each sector to locate at the center of the group of CP-associated RSs (Figure 3, A–C). An analysis of 56 cross-section images revealed that the CP surface had two preferred sites for association with the RS: one near the C1a and the other near the C1b projection. These two preferred sites must bind to the spokeheads more strongly than the other regions of the CP.

Previous studies showed that the *Chlamydomonas* CP, when released from the axoneme, forms a helical complex; when contained in the axoneme, it must be forced to assume a straight form with a 360 degree twist per the length of the flagellum [40], [41]. This tendency of the CP to assume a helix might bias the distribution of the apparent spoke-interaction sites in the 10- or 11-doublet axonemes (Figure 3, B and C). To address this possibility, we examined the helical tendency of the CP in the spoke-less *pf14* axonemes, in which the CP should assume a small-amplitude helical form facing its outer surface to the outer doublet wall (Figure 3, D and E). An analysis of 80 cross sections of *pf14* axonemes indicated that the C1 microtubule is located outer side of the CP helix, i.e. closest to the doublet wall. This distribution pattern is clearly different from the pattern in the axonemes of *bld12*, which showed two preferred orientation regions (Figure 3, B–E). We therefore concluded that the helical tendency of the CP did not mask the CP orientation resulting from its interaction with RSs.

### Removal of CP projections identifies multiple weak CP-spoke association sites

**Figure 4:**
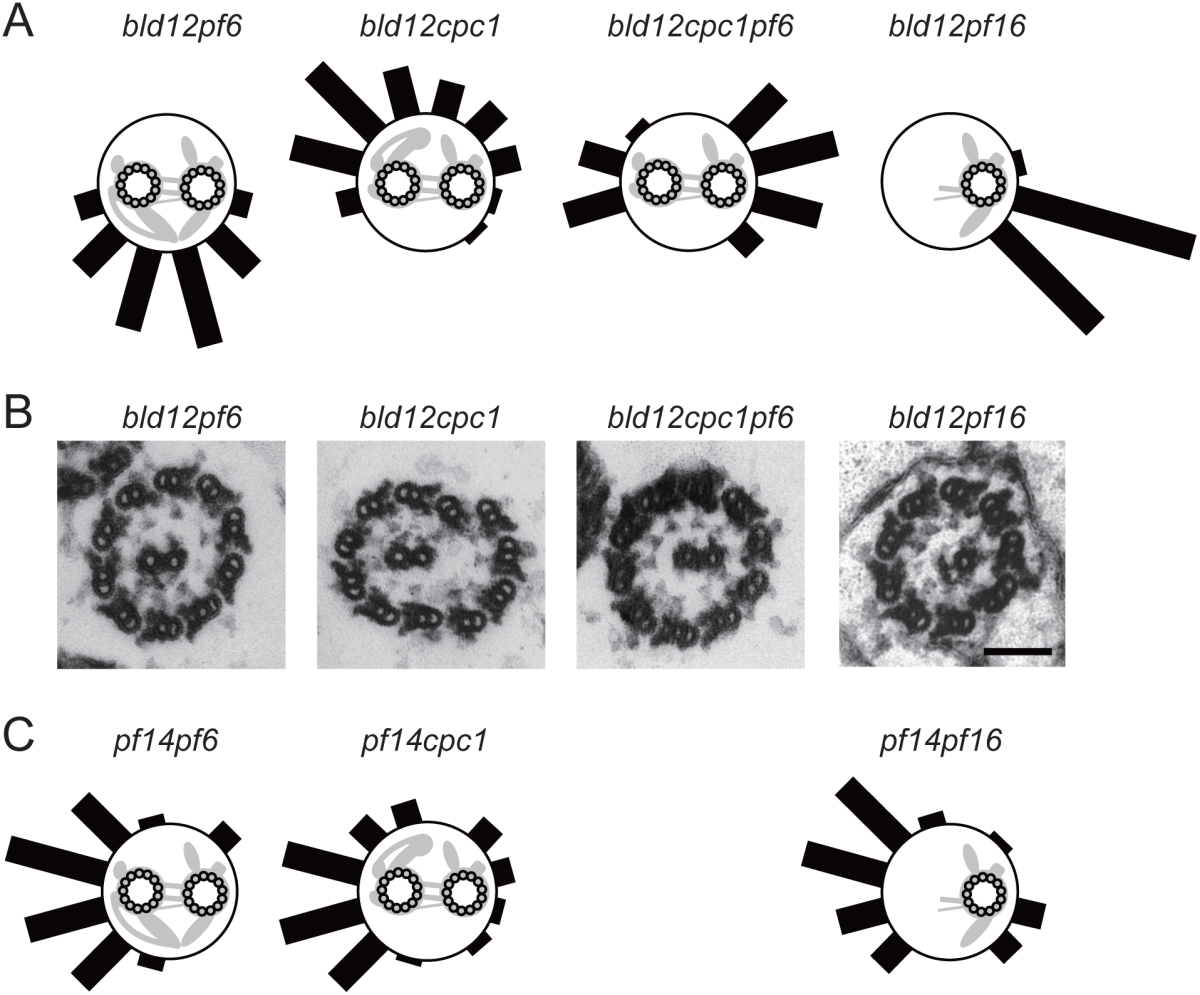
Removal of CP projections manifests weak CP-spokehead association. (A) Distributions of the spokehead-interaction sites on the CP in cross section images of 10- or 11-doublet axonemes of *bld12pf6*, *bld12cpc1*, *bld12cpc1pf6*, and *bld12pf16* (n = 59, 49, 40, and 41), which lack specific CP projections. (B) Cross section images that represent the peaks in the histograms in (A). (C) Distributions of contact sites on the CP surface with the outer doublet wall in cross section images of *pf14pf6*, *pf14cpc1*, and *pf14pf16* (n = 33, 69, and 46). The differences between the distributions of *bld12pf6* and *pf14pf6*; between *bld12cpc1* and *pf14cpc1*, and between *bld12pf16* and *pf14pf16*, are significant (χ^2^ test, p<0.05 for each of the three pairs). These distribution patterns are similar to the pattern in *pf14*.

We next examined CP-RS interactions in double mutants between *bld12* and each one of four mutants that lack specific CP projections (Figure 4). The CP mutants used were *pf6* lacking the prominent projection C1a [42]; *cpc1* lacking C1b and C2b [35]; *pf6cpc1* double mutant lacking these three projections; and *pf16* lacking the C1 microtubule [42]. Interestingly, the spokeheads preferentially bound to the C1b–C2b region when the C1a projection was absent, whereas they bound to the C1a–C2a region when C1b and C2b were absent. In *pf6cpc1* or *pf16*, the spokeheads tended to bind to the C1d and C2c regions, or to the C2b region, where binding was only infrequently observed in *bld12* axonemes (Figure 4, A and B). As controls, we also analyzed the axonemal images of double mutants *pf14pf6*, *pf14cpc1*, and *pf14pf16* because we were particularly concerned that the mutant CPs might have varied tendencies to assume helical forms, which might affect the results of our analysis. The histograms obtained in the control experiments showed that the helical tendency was not largely affected by the *pf6*, *cpc1*, or *pf16* mutations (Figure 4C). For example, the *pf14pf16* CP, which lacks the C1 microtubule, displayed the same tendency as the *pf14* CP (Figure 3D); that is, the side of the C2 that would be positioned closest to the C1 in the wild type CP was still positioned on the outside of the helix. These results suggest that, although different regions of the CP surface differ in the spokehead binding affinity, almost the whole area of the CP surface can bind to the spokeheads.

## Discussion

### Formation of the CP

We showed that the CP did not assemble in 8-doublet axonemes of *bld12*, but assembled in those of *bld12pf14* lacking the RS. Furthermore, while 9-doublet axonemes of *bld12pf14* only rarely contained two CPs, its 10-doublet axonemes frequently contained two CPs (Figure 2, Table 1). Formation of two CPs has previously been observed in flagella and cilia that lack the RS: for example, flagella of *Chlamydomonas pf14* and *pf14pf6cpc1* mutants [20], [23]; and nodal cilia of rabbit [43]. These observations suggest that CP assembly depends on the internal space of the axoneme. Our results, demonstrating a clear correlation between the CP assembly and the space size, lend strong support to the previous proposal (Table 1).

The distal end of the CP in *Chlamydomonas* flagella and *Tetrahymena* cilia is capped by a plate-like structure that is indirectly attached to the membrane through a spherical bead. In contrast, the proximal end of the CP, located near the transition zone, is associated with no detectable structure [12], [13]. In some *Chlamydomonas* mutants with defects in the transition zone, as well as in isolated tracheal epithelial cells, the CP microtubules grow proximally into the centriole, suggesting that the proximal end is not the site of nucleation for CP growth [16], [17]. Lechtreck et al. (2013) reported that, when gametes of a *Chlamydomonas* mutant lacking the CP are mated with wild type gametes, the mutant flagella in the fused cells start to produce the CP at the middle portion of the axoneme. This observation indicates that no organizing center is required for the CP assembly at either end of the flagellum. These authors also observed that the RS-lacking *pf14* axonemes contain two CPs (four microtubules) with correct polarity. Together with their findings, our observation of the extra CP in *bld12pf14* implies that the space size within the axoneme is an important factor that directs CP formation in the axoneme. Possibly, when the CP precursors are present in the axoneme, the CP may form spontaneously without interacting with any template or RSs. In such a case, only the available space and the amount of the precursors may limit the number of the CPs produced.

### Nature of CP-RS interaction for the regulation of flagellar motility

A minor population of *bld12* axonemes having 10 or 11 outer doubles exhibited distortion in the circular arrangement of the outer doublets. This observation clearly indicates that the RS binds to the CP and the binding exerts force. Previous studies have provided substantial evidence that the CP and RS form a signal transduction pathway that modulates dynein activity through phosphorylation of a specific subunit of inner-arm dynein [44]–[46]. The RS is likely to chemically and mechanically control the dynein activity based on the interaction between the CP projections and the spokeheads [9], [21]. However, whether or not the RS exerts mechanical force on the outer doublet has been unknown. Our present study is the first to show that RSs actually pull the outer doublet microtubules toward the axonemal center. Such a mechanical force may be at the center of the regulatory function of the CP/RS.

The CP-RS interaction should be transient and the binding strength is weak enough to allow such an interaction. This is because the *Chlamydomonas* CP rotates within the axoneme [40], [41] and the RS slides over the CP for a certain distance as the axoneme propagates bending waves [47]. In this study, however, we showed that the interaction is still strong enough to distort the circular arrangement of the outer doublets. This finding prompts us to speculate that the mechanical force transmitted by the RS could change the relative position of the doublet microtubules and dyneins, and thereby transiently activate or inactivate the dyneins located in a particular region of the axoneme. A change in the dynein-microtubule positioning has also been postulated in the geometric clutch model of Lindemann [48]. The location of dynein molecules activated or inactivated by the CP/RS should propagate along the axoneme as the twisted CP rotates. In the cilia and flagella of multicellular organisms and some unicellular organisms, the CP neither assumes a helical shape nor rotates in the axoneme although sharing the structure and components with the *Chlamydomonas* CP [23], [49], [50]; in those organisms, the stationary CP determines the plane of axonemal beating possibly by activating dynein molecules on a particular side of the axoneme [51], [52].

Our image analyses of mutant axonemes suggest that, while most area of the CP surface can bind to the RS, the two major projections on the C1 microtubule bind stronger than the other projections on the C2 microtubule. This asymmetric distribution of the RS binding affinities on the CP surface must be important for the signal generation by the rotating CP. Although the present study does not provide information as to whether the stronger binding promotes or inhibits dynein-driven microtubule sliding, previous studies suggest that the C1 microtubule or its projections enhances microtubule sliding in axonemes [46], [53]–[55]. Thus the doublet-pulling by stronger RS binding to the C1 surface may activate dyneins and promote microtubule sliding.

Our results are in apparent contradiction with a non-specific CP-RS interaction model recently proposed by Oda et al. (2014). These authors showed that the flagella of the *pf6* mutant lacking the C1a projection recovered motility if any one of three protein tags (hemagglutinin, biotin carboxyl carrier protein, and green fluorescent protein) was attached on top of the spokehead. Because the extent of motility recovery increased in the order of the size of the tag, they proposed that the added tag elongated the RS and compensated the loss of the C1a projection, possibly by enabling RSs to collide with the CP. The proposed physical CP-RS interaction must be non-specific since the three protein tags used are structurally unrelated. In contrast, our analysis of 10- or 11-doublet axoneme images showed that the C1a projection, together with the C1b projection, preferentially associates with the RSs among all the CP projections, favoring the view that the C1a projection pulls the outer doublet in a fairly specific manner. Both their results and our results must reflect some aspects of the CP-RS interaction, but their relationship is not understood. The molecular mechanism of the CP-RS interaction remains one of the most interesting problems in cilia/flagella motility studies.

An obvious question regarding the present study is whether or not the axonemes with aberrant numbers of outer doublets are motile. We may imagine that 8-doublet flagella are non-motile because they lack the CP, like the flagella of non-motile mutants such as *pf18* and *pf19* [56]–[58]. However, it is difficult to predict whether or not axonemes with 10 or 11 doublets can display some motility. Development of techniques that determine the number of axonemal microtubules under the microscope, or those that permit constant production of flagella with 10–11 doublets, may well provide answers. We can hope that the answer will provide a strong clue as to why motile cilia and flagella almost always contain nine outer doublets.
